# Genome-wide annotation and analysis of zebra finch microRNA repertoire reveal sex-biased expression

**DOI:** 10.1186/1471-2164-13-727

**Published:** 2012-12-26

**Authors:** Guan-Zheng Luo, Markus Hafner, Zhimin Shi, Miguel Brown, Gui-Hai Feng, Thomas Tuschl, Xiu-Jie Wang, XiaoChing Li

**Affiliations:** 1 State Kay Laboratory of Plant Genomics, Institute of Genetics and Developmental Biology, Chinese Academy of Sciences, Beijing, 100101, China; 2 Laboratory of RNA Molecular Biology, The Rockefeller University, New York, NY, 10065, USA; 3 Neuroscience Center of Excellence, Louisiana State University Health Sciences Center, New Orleans, LA, 70112, USA

**Keywords:** Zebra finch, miRNAs, Sequence variations, Tissue-enriched miRNA expression, Z chromosome, Sex-biased miRNA expression

## Abstract

**Background:**

MicroRNAs (miRNAs) are small noncoding RNAs that regulate gene expression post-transcriptionally in a wide range of biological processes. The zebra finch (*Taeniopygia guttata*), an oscine songbird with characteristic learned vocal behavior, provides biologists a unique model system for studying vocal behavior, sexually dimorphic brain development and functions, and comparative genomics.

**Results:**

We deep sequenced small RNA libraries made from the brain, heart, liver, and muscle tissues of adult male and female zebra finches. By mapping the sequence reads to the zebra finch genome and to known miRNAs in miRBase, we annotated a total of 193 miRNAs. Among them, 29 (15%) are avian specific, including three novel zebra finch specific miRNAs. Many of the miRNAs exhibit sequence heterogeneity including length variations, untemplated terminal nucleotide additions, and internal substitution events occurring at the uridine nucleotide within a GGU motif. We also identified seven Z chromosome-encoded miRNAs. Among them, miR-2954, an avian specific miRNA, is expressed at significantly higher levels in males than in females in all tissues examined. Target prediction analysis reveals that miR-2954, but not other Z-linked miRNAs, preferentially targets Z chromosome-encoded genes, including several genes known to be expressed in a sexually dimorphic manner in the zebra finch brain.

**Conclusions:**

Our genome-wide systematic analysis of mature sequences, genomic locations, evolutionary sequence conservation, and tissue expression profiles of the zebra finch miRNA repertoire provides a valuable resource to the research community. Our analysis also reveals a miRNA-mediated mechanism that potentially regulates sex-biased gene expression in avian species.

## Background

MicroRNAs (miRNAs) are short non-coding RNA molecules that regulate gene expression post-transcriptionally [1]. miRNAs are transcribed by RNA polymerase II. Primary transcripts of miRNAs are cleaved in the nucleus by the nuclease Drosha to generate precursor miRNAs (pre-miRNAs) with a characteristic hairpin-like secondary structure [2-4]. The pre-miRNAs are then exported to the cytoplasm and further cleaved by the RNase III enzyme Dicer to release a 21–23 nt small RNA duplex from the stem region of the hairpins [2,5-7]. In most cases, only one strand of the small RNA duplex is retained as mature miRNA, and the other strand, termed miRNA star (miRNA*), is degraded [8]. In some cases, however, both strands from the same hairpin are retained as functional miRNAs, which are annotated as miRNA-5p and miRNA-3p, respectively, depending on which arm of the hairpin they are derived [9]. Sequence variations, such as nucleotide deletion, insertion, untemplated terminal extension, and editing, have been observed for some miRNAs [10-13]. The majority of animal miRNAs function by binding to the 3^′^-untranslated regions (UTRs) of target mRNAs via sequence complementarity to induce mRNA degradation or repress protein translation [14-16]. miRNAs have been found in most eukaryotes and many of them are conserved through evolution. The recent application of deep sequencing technology has also revealed an increasing number of species-specific miRNAs [10,17-21]. It is estimated that over 60% of human protein-coding genes might be regulated by miRNAs [22]. By fine-tuning gene expression, miRNAs regulate many biological processes ranging from cell proliferation, cell fate specification, cell differentiation, apoptosis, animal development, metabolism, to various disease conditions [1,16,23].

The zebra finch (*Taeniopygia guttata*), an oscine songbird, provides a unique animal model for neurobiological research with its characteristic learned vocal behavior [24]. Zebra finches use a discrete set of interconnected brain nuclei and pathways, commonly referred to as the song system, to control song behavior [25]. Song behavior in zebra finches is sexually dimorphic: only male birds sing, and the song system is highly developed in males [26,27]. For decades, zebra finches have been widely used to study vocal learning, neuronal replacement, and sexually dimorphic development of male and female brains [24,28]. In recent years, large efforts have been devoted to developing genomic resources and extending research in zebra finches to molecular and genomics levels [29-35]. As part of this effort, we have systematically characterized miRNA expression in various tissues of adult female and male zebra finches using the Illumina high throughput sequencing platform. Taking advantage of the large sequence dataset now available, we analyzed features of these miRNAs, including miRNA sequence conservation through evolution, miRNA clusters, tissue-enriched expression, and sequence variations. Previously, using the 454 sequencing platform, we detected a Z chromosome encoded miRNA, miR-2954, in the male zebra finch brain [32]. This miRNA has recently been shown to be expressed in the auditory forebrain of zebra finches, and its expression is regulated by song exposure [35]. We have further explored the expression of miR-2954 and other Z chromosome encoded miRNAs in male and female tissues and have found that miR-2954 is predominantly expressed in all male tissues examined. Our target prediction analysis revealed that miR-2954, but not other Z chromosome encoded miRNAs, preferentially targets Z chromosome encoded genes.

## Results

### General features of zebra finch miRNAs

**Figure 1:**
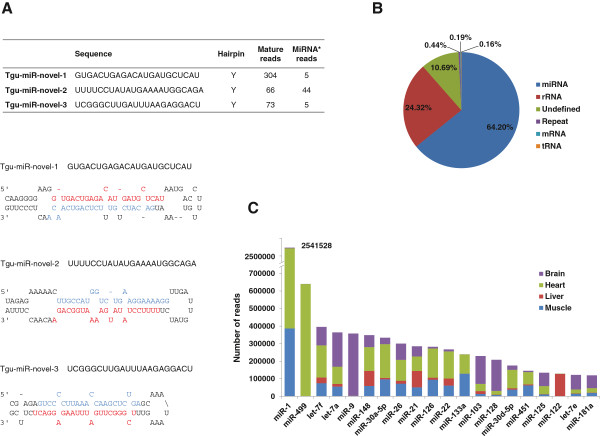
**miRNA annotation and expression analysis in zebra finch. **(**A**) Mature sequences, expression counts, and precursor sequences with predicted hairpin-like secondary structures of three novel miRNAs identified in the zebra finch. Nucleotides labeled in red and blue in the precursor sequences represent mature miRNAs and their star sequences, respectively. The read numbers are combined from four tissues and two sexes. (**B**) Relative percentages of small RNA populations among the total sequence reads. Calculations were based on the total reads of the eight libraries combined. (**C**) The 20 most abundantly expressed miRNAs.

We prepared eight small RNA libraries from four tissues – brain, heart, liver, and muscle – of adult female and male zebra finches. These libraries were sequenced using the Illumina Genome Analyzer II high throughput sequencing platform. We obtained a total of 23,366,676 raw sequence reads from all libraries combined. After adaptor trimming and removal of low quality reads and orphan sequences (single reads), 19,424,182 high quality sequence reads were retained for subsequent analysis (Additional file 1). Of these reads, 60% mapped perfectly to the zebra finch genome assembly (release 1.0). We extracted flanking sequences around mapped reads and used mFold [36] to search for hairpin-like secondary structures. A total of 169 mature miRNAs were identified, which exhibited good hairpin-like precursor structures and matched known miRNAs recorded in miRBase (version 17.0) with high sequence homology (identical or with one mismatch). Considering that the current zebra finch genome assembly is relatively new and may contain gaps [32], we compared the unmapped sequence reads to known miRNA sequences in miRBase, and identified 21 additional miRNAs with high sequence homology (identical or with one mismatch) to known miRNAs in other species. Following the general criteria for miRNA annotation [37], we identified three novel zebra finch specific miRNA candidates, which are supported by good hairpin-like precursor structures, presence of corresponding star sequences, and relatively high expression (Figure 1A). Taken together, we identified 193 distinct zebra finch miRNAs. The sequences, genomic locations, and relative expression levels of these miRNAs are summarized in Additional file 2.

miRNAs and their variants accounted for ~64% of all high quality reads. The remaining sequence reads were classified as rRNA/tRNA fragments, repeat-associated small RNAs, degradation products of mRNAs, and undefined small RNAs (Figure 1B). The expression levels of individual miRNAs spanned a wide range, from a few copies to thousands of copies (Additional file 2). For example, tgu-miR-1, which was highly expressed in both heart and muscle, was represented by 2,541,528 reads, accounting for 25% of the total miRNA reads (Figure 1C), whereas the three novel zebra finch miRNAs were expressed at relatively low levels, represented by 304, 66, and 73 reads respectively (Figure 1A). (Note, these numbers represent combined totals from four tissues and two sexes. For read counts in individual tissues and sexes, see Additional file 2). All of the top 20 most abundantly expressed miRNAs were conserved in vertebrates, and they comprised 76% of the total miRNA sequence reads (Figure 1C).

### Potential avian specific miRNAs

**Figure 2:**
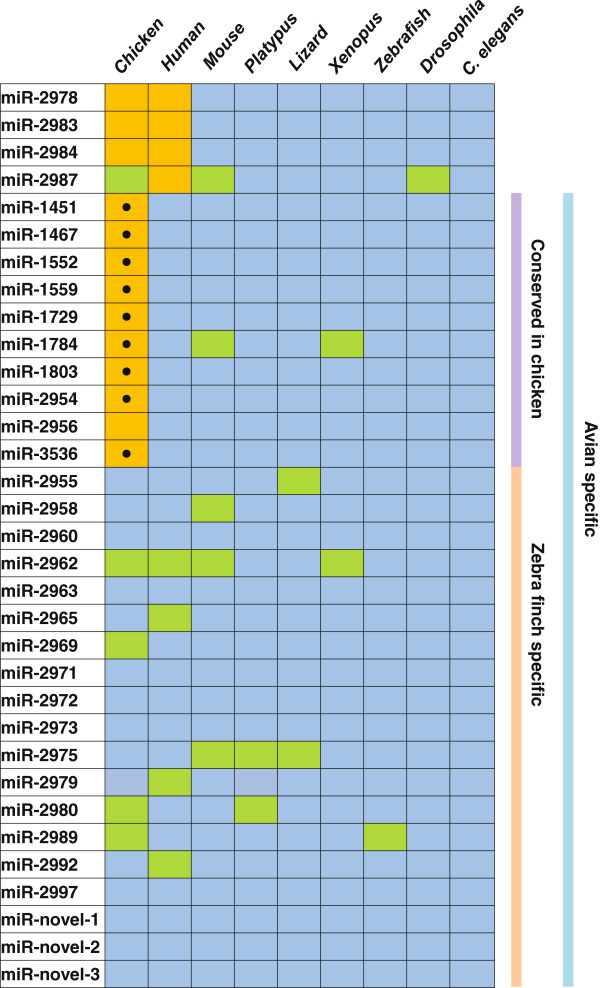
**Conservation status of avian-specific miRNAs in nine animal species.** The orange color indicates miRNA sequence homologs with predicted hairpin-like structures were found in a genome by our analysis. Black dots indicate homolog miRNAs are already recorded in miRBase as known miRNAs. Green color indicates mature miRNA sequences, but not precursor sequences, were found in a genome, and they were not counted as having homologs in other genomes. Blue is the background. Note, sequence homologs for miR-2978, miR-2983, miR-2984, miR-2987, and miR-2956 were found in the chicken genome, but they were not recorded in miRBase, so they didn′t get a black dot. We reported zebra finch specific miRNAs (miR-2955 through miR-2997) previously [32], and they are already in miRBase, so they were not considered to be novel miRNAs.

By comparing to mature miRNA sequences in miRBase, 33 of the 193 zebra finch miRNAs did not have homologs (identical or with one mismatch) outside of avian species. To investigate whether these 33 miRNAs had unidentified homologs in other genomes, we searched for homologous sequences in the genomes of 9 animal species including *C. elegans*, drosophila (*D. melanogaster*)*,* zebrafish (*Danio rerio*), X. tropicalis *(Xenopus tropicalis)*, lizard (*Anolis carolinensis*), platypus (*Ornithorhynchus anatinus*), chicken (*Gallus gallus*), mouse (*Mus musculus*), and human (*Homo sapiens*). Sequence homologs of 4 miRNAs (miR-2978, miR-2983, miR-2984, and miR-2987) were found in at least one non-avian vertebrate, and these were subsequently excluded from the list of avian-specific miRNAs. Thus, 29 miRNAs were classified as avian-specific miRNAs, of which 19 were zebra finch specific (Figure 2). By extending this analysis to all the 193 zebra finch miRNAs, we found that 37 miRNAs were conserved from *C. elegans* through humans, 103 were conserved in vertebrates, and 24 were only conserved between avian and mammals (Additional file 3).

### miRNA clusters

Using a distance of 10 kb between any two miRNA genes as a cutoff, 87 miRNA genes were grouped into 36 genomic clusters. We named these miRNA gene clusters according to the name of the first member of each cluster followed by the number of miRNA genes within the cluster in parenthesis, e.g., cluster *tgu-mir-24(3)* contains *tgu-mir-24, tgu-mir-27b,* and *tgu-mir-23b* (Additional file 4). Among these clusters, 3 appeared to be formed by tandem duplications of a single miRNA gene, e.g., the *tgu-mir-2989(2)* cluster on chromosome 8 contains 2 copies of the *tgu-mir-2989* gene (Additional file 4). A majority of clusters were conserved in vertebrates (having more than 2 miRNA genes in the same cluster in other species); one cluster (*tgu-let-7a-2(3)*) was also conserved in *D. melanogaster*, but none was conserved in *C. elegans*. One cluster (*tgu-mir-2989(2))* appeared to be zebra finch specific.

### miRNA*

We found 150 miRNA* sequences for 131 mature miRNAs (Additional file 5). For miRNAs encoded by multiple genomic loci, a single mature sequence can have more than one star sequence, presumably originating from different precursors. For example, three different star sequences were detected for tgu-miR-7, which has three genomic loci. These three tgu-miR-7* sequences had different read counts, indicating that the three *tgu-miR-7* genes were transcribed with different promoter activities and/or different efficacies of precursor processing (Additional file 6). Most of the star sequences were detected at considerably lower levels compared to their respective dominant strands. Yet, for 16 miRNAs, both strands were detected at comparable levels (< 10 fold differences, Additional file 7), suggesting that both strands may function as mature miRNAs. The relative read counts for the two strands of these miRNAs were similar across the four tissues examined (Additional file 5), indicating that the mechanisms retaining both strands were tissue independent. Interestingly, for several miRNAs (e.g., tgu-miR-142, tgu-miR-214, and tgu-miR-455), both strands have been detected at similar levels in mice and chickens as well [12,38], suggesting a functional conservation of these star sequences in vertebrates.

### miRNAs generated by atypical biogenesis pathways

Mirtrons are a special group of miRNAs that are derived from short intronic sequences by splicing machinery rather than by Drosha cleavage [39-41]. Recently, Glazov and colleagues reported the identification of 12 mirtrons in chickens [38]. We did not, however, find sequence homologs of any of the chicken mirtrons in our miRNA set, nor did we find any miRNAs mapping to either boundaries of a short intron. This discrepancy might be partly explained by the fact that the chicken mirtrons were detected in embryos and exhibited low copy numbers [38], whereas our miRNAs were from adult tissues. Alternatively, it may simply be a consequence of the incomplete annotation of intronic regions in the zebra finch genome.

miR-451 is a vertebrate miRNA whose maturation depends on Ago2 cleavage rather than the common Dicer pathway [42,43]. We found that *tgu-miR-451* was located on chromosome 19, about 100 nt downstream from miR-144, and the mature and precursor sequences of tgu-miR-451 were highly conserved with those in chickens, mice, and humans. Similar to its mammalian counterparts, the 5′-end of tgu-miR-451 was well defined, whereas its 3′-end was highly variable, extending into the loop region of the hairpin structure, presumably reflecting imprecise cleavage by Ago2 (Additional file 8).

### miRNA expression patterns in tissues

**Figure 3:**
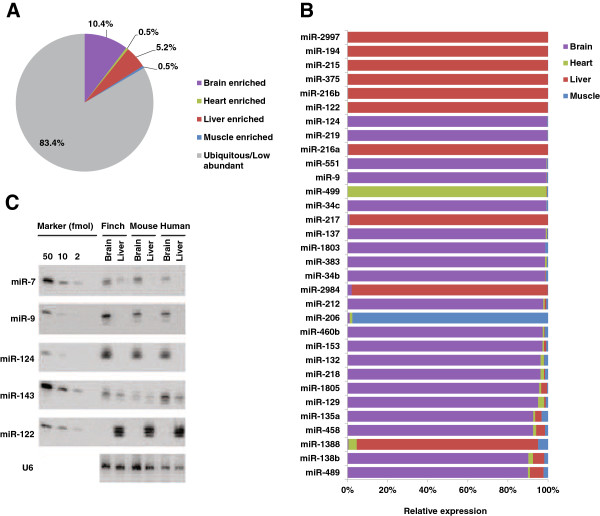
**Tissue enriched expression of miRNAs. **(**A**) Summary of percentages of miRNAs with enriched expression in each tissue. (**B**) miRNAs with the most tissue-enriched expression. Note, calculations in A and B were based on reads combined from both sexes. (**C**) Northern blot analysis showing the expression of miR-7, miR-9, miR-124, miR-143, and miR-122 in the brain and liver tissues of zebra finches, mice and humans.

We next examined the expression patterns of zebra finch miRNAs in brain, heart, liver, and muscle. Overall, the brain displayed the most diverse miRNA expression and had the largest number of tissue-enriched miRNAs (Figure 3A). About 17% of all miRNAs (32 of 193) showed enriched expression in a single tissue, and two thirds of them (20 of 32) were highly enriched in brain based on combined read counts from males and females (Figure 3A and 3B). However, the two most abundantly expressed miRNAs (miR-1 expressed in heart and muscle and miR-499 in heart) showed little or no expression in brain. Since the sequences of many miRNAs are conserved through evolution, we asked whether the tissue-enriched miRNA expression patterns were also conserved. To this end, we analyzed previously published data from human, mouse, and sea slug (*Aplysia)*[10,19]. Seven miRNAs (miR-9, miR-124, miR-137, mir-153, miR129, mir-218, and miR-138b) showed brain-enriched expression in both humans and zebra finches, and miR-124, miR-137, miR-153, and miR-34b have also been detected in the *Aplysia* nervous system (Table 1). We validated the conservation of tissue expression patterns of five miRNAs in human, mouse, and zebra finch by Northern blot analysis (Figure 3C).

**Table 1:** Conservation analysis of brain enriched miRNAs in three other species

**miRNAs**	**Total reads**	**Reads in brain**	Conservation*		
			**Human**	**Mouse**	***Aplysia***
miR-9	357811	354947	√	√	
miR-124	111628	111170	√	√	√
miR-137	28008	27599	√	√	√
miR-153	16599	16188	√		√
miR-212	8361	8168			
miR-135a	7451	7053			
miR-219	7228	7198			
miR-132	6496	6172			
miR-34b	6401	6322			√
miR-129	5909	5535	√		
miR-551	4992	4949			
miR-218	4221	4024	√	√	
miR-383	3822	3743			
miR-34c	3628	3593			
miR-460b	1447	1402			
miR-458	1013	958			
miR-138b	386	358	√	√	
miR-1805	306	297			
miR-489	304	285			
miR-1803	264	261			

* √ indicates brain-enriched expression in the examined species.

miRNAs within a genomic cluster tend to exhibit parallel relative expression patterns across tissues, probably reflecting their shared promoter and tissue specific cistronic transcriptional control. However, the expression abundance of different members of a cluster in a given tissue can be drastically different. For example, miR-133a and miR-1 of the *133a(2)* cluster were both expressed in heart and muscle, but the expression of miR-1 was about 10 times higher than that of miR-133a in each of these tissues. This difference in expression levels between members was characteristic of many genomic clusters (Additional file 9), suggesting that a precursor specific event during the miRNA maturation process might regulate the expression levels of individual miRNAs in a genomic cluster.

### Sequence variations

**Figure 4:**
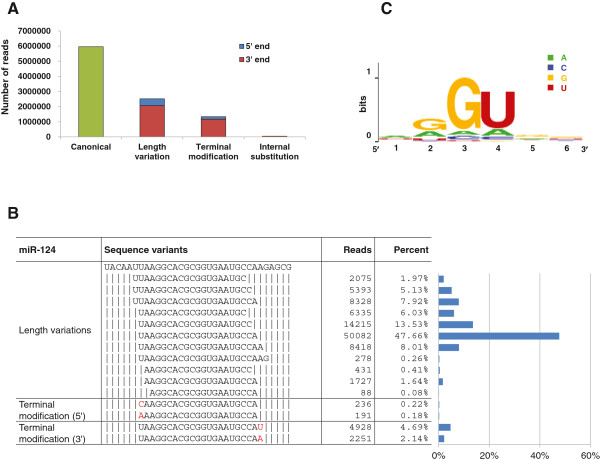
**miRNA sequence variants. **(**A**) Distribution of sequence variation types among all miRNA reads. (**B**) Sequence variants in miR-124. The nucleotides differing from the template genome are highlighted in red. Blue bars to the right indicate frequencies of these variants. (**C**) Motifs identified among internal substitution sites. The overall height of a stack indicates sequence conservation at that position: the higher the stack, the more the position is conserved. The heights of nucleotides within a stack indicate the relative frequency for each nucleotide at that position. We analyzed 6-nucleotide sequences containing an internal substitution site having a substitution rate > 5%, and found that a GGU motif was preferentially present among all sequences. The numbers (1–6) below the X axis indicate nucleotide positions in the motifs.

Taking advantage of the large sequence dataset, we analyzed miRNA sequence variations. We classified miRNA isoforms into three major groups: length variations, untemplated terminal nucleotide additions, and internal substitutions (Figure 4A). The length variants accounted for 25% of the total miRNA reads, a majority of which (> 80%) were 3' variants (Figure 4A). This is in good agreement with observations in other species, further supporting the notion that precision at cleavage events at the 5'-termini is necessary to protect the seed sequence at positions 2–8 of the mature miRNA [12,44,45]. Nonetheless, the read numbers of 5' offset isoforms of several miRNAs were relatively high. For example, miR-124, a brain enriched miRNA, had several 5' offset isoforms, with the combined reads accounting for 15% of all reads (Figure 4B). Another prominent example was miR-133a, which had two main 5'-isoforms, miR-133a1 and miR-133a2, of which the 5' terminus of miR-133a2 was shifted 1 nucleotide in the 3' direction. These two isoforms were each represented by 77,642 and 98,840 reads, accounting for 32% and 41% of the total reads. Interestingly, similar patterns of 5' heterogeneity are also observed in mouse miR-124 and miR-133a [12,46], indicating that the alternative processing mechanisms giving raise to these isoforms might be evolutionarily conserved.

Untemplated nucleotide additions to miRNA 3' ends are observed for many miRNAs in worms, flies, and mammals [10,12,44,45]. Similar to those observed in other species, nucleotides most frequently added to zebra finch miRNAs were U and A, with the U additions occurring more frequently than the A additions (66 miRNAs had U additions and 35 miRNAs had A additions, Additional file 10). For some miRNAs, the extended isoforms outnumbered the canonical forms. For example, miR-456 had 7 fold more extended reads than non-extended reads, and miR-24 had 23,754 extended reads compared to 7,212 non-extended reads (Additional file 10). We also observed that A addition had a greater tendency to occur on 5p arms, occurring 26 times on 5p arms compared to 9 on 3p arms, while U addition occurred slightly more often on 3p arms, occurring 36 times on 3p arms compared to 30 on 5p arms. Compared with previously published data, it appeared that patterns of untemplated extension of some miRNAs were conserved among multiple vertebrate species. For example, the A extensions of miR-99 and miR-101 and the U extensions of miR-15a, miR-24, miR-106, miR-124, and miR-425-5p were observed in zebra finch, human, and mouse [13,46].

In mammals, miRNA editing events in which adenosine is converted to inosine by adenosine deaminases (ADARs) are observed for several miRNAs; the resultant inosine is detected in sequencing as an A-to-G conversion [10,12,47-49]. Among our data, however, the A-to-G change was found in just ~0.3% of total miRNA reads in the brain library and in all 8 libraries combined. This frequency does not differ significantly from the sequencing error rate observed among our synthetic internal control sequences spiked into library preparations (<0.5%). We also searched for A-to-I editing events at specific sites in individual miRNAs. Using a criterion of >5% mismatch frequency, only tgu-miR-24 exhibited significant A-to-G change at the 15th position of the mature sequence. This change occurred in all tissues, but had the highest frequency in brain (~9%, Additional file 11). We next examined all miRNAs that had any internal nucleotide change. Thirty-two miRNAs displayed internal nucleotide changes with a frequency >5%. Nucleotide changes at the U position within a GGU motif were the most frequent substitution (25 out of the 32), and it appeared that substitution with any of the other three nucleotides was permissible (Figure 4C). For several miRNAs in this group, the GGU motifs occurred within the seed sequence (Table 2); thus, substitution of the U nucleotide could potentially alter miRNA targeting specificity (Table 2).

**Table 2:** miRNAs having substitutions at the GGU motif

**miRNAs**	Canonical sequence*	**Modified reads**	**Percentage**
tgu-let-7a	UGA**GGU**AGUAGGUUGUAUAGUU	11500	5.5%
tgu-let-7b	UGA**GGU**AGUAGGUUGUGUGGUU	1629	6.7%
tgu-let-7c	UGA**GGU**AGUAGGUUGUAUGGUU	1556	5.2%
tgu-let-7d	AGA**GGU**AGUAGGUUGCAUAGUU	395	5.5%
tgu-let-7e	UGA**GGU**AGUAGAUUGAAUAGUU	3864	5.2%
tgu-let-7f	UGA**GGU**AGUAGAUUGUAUAGUU	12490	5.1%
tgu-let-7i	UGA**GGU**AGUAGUUUGUGCUGUU	3792	8.2%
tgu-miR-122	UGGAGUGUGACAAU**GGU**GUUUG	6211	14.0%
tgu-miR-133b	UU**GGU**CCCCUUCAACCAGCUAU	18	5.1%
tgu-miR-140	ACCACAG**GGU**AGAACCACGGAC	674	5.0%
tgu-miR-15a	UAGCAGCACAUAAU**GGU**UUGU	382	5.4%
tgu-miR-15c	UAGCAGCACAUCAU**GGU**UUGU	187	5.4%
tgu-miR-181b	AACAUUCAUUGCUGUC**GGU**GGGU	254	6.2%
tgu-miR-183	UAUGGCACU**GGU**AGAAUUCACU	5	5.1%
tgu-miR-18a	UAA**GGU**GCAUCUAGUGCAGAUA	228	12.5%
tgu-miR-18b	UAA**GGU**GCAUCUAGUGCAGUU	7	5.0%
tgu-miR-196	UA**GGU**AGUUUCAUGUUGUUGGG	8	10.0%
tgu-miR-221	AGCUACAUUGUCUGCUG**GGU**UUC	2636	6.4%
tgu-miR-222	AGCUACAUCUGGCUACUG**GGU**CUC	1413	6.5%
tgu-miR-2970	GACAGUCAGCAGUU**GGU**CUGG	219	11.7%
tgu-miR-363	AAUUGCAC**GGU**AUCCAUCUGU	83	6.8%
tgu-miR-383	CAGAUCAGAA**GGU**GAUUGUGGC	101	7.3%
tgu-miR-456	CAGGCUGGUUAGAU**GGU**UGUC	34	7.8%
tgu-miR-458	AUAGCUCUUGGAAU**GGU**UCUGC	31	5.1%
tgu-miR-551	GCGACCCAUACUU**GGU**UUCAG	69	5.0%

*The seed regions (2–8th nucleotides) are underlined. The GGU motifs are marked in boldface.

### Male-biased expression and targeting of miR-2954

**Figure 5:**
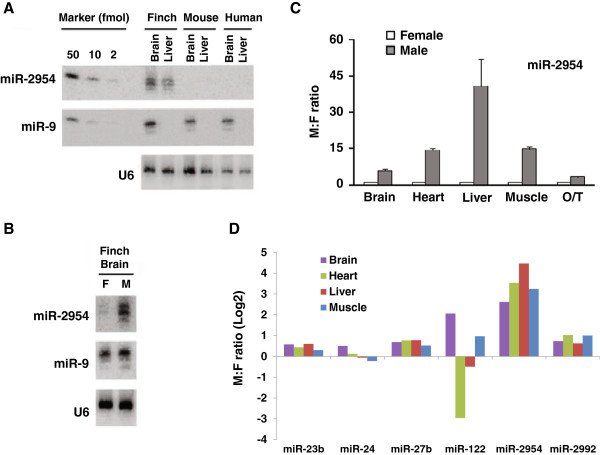
**Expression analysis of miR-2954. **(**A**) Northern blot analysis shows that miR-2954 was expressed in brain and liver tissues of male zebra finches, but was not detectable in mouse or human tissues. (**B**) Northern blot shows expression of miR-2954 and miR-9 in the brain tissues of female and male zebra finches. (**C**) Sex-biased expression of miR-2954 in the brain, heart, liver, muscle, ovary, and testis of male and female zebra finches validated by qRT-PCR. (**D**) Expression ratios of Z chromosome-encoded miRNAs between male and female zebra finch tissues based on sequence reads. Note, miR-2973, another Z chromosome-encoded miRNA, was not included here because its total combined reads were < 100.

miR-2954 is a recently identified avian miRNA, which has a single genomic locus on the zebra finch Z chromosome [32,35]. We did not detect it in the chicken genome assembly (version galGal3) or in the genomes of other animal species (Figure 2). However, we found the mature miR-2954 sequence among chicken ESTs, and its expression in chicken embryo is detected by Northern blot analysis [50]. Tgu-miR-2954 was expressed in a sex-biased manner in all four examined tissues; and its expression was significantly higher in male tissues than in female tissues (Additional file 2). We validated this expression pattern by Northern blot analysis and quantitative real-time PCR (qRT-PCR) (Figure 5A, B, and C). Our original sequencing samples did not include ovary and testis; however, using qRT-PCR, we found that the expression of miR-2954 was 3-fold higher in testis than in ovary (Figure 5C). In addition to miR-2954, six other miRNAs (miR-23b, miR-24, miR-27b, miR-122, miR-2973, and miR-2992) were encoded solely by the Z chromosome, and most of them showed slightly higher expression in male tissues than in female tissues (Figure 5D). The one exception was miR-122, which was expressed at a higher level in heart in females than in males. Several miRNAs (miR-7, miR-9, miR-101, and miR-204) that are encoded by both the Z and the A chromosomes did not exhibit consistent sex-biased expression (Additional file 12).

**Figure 6:**
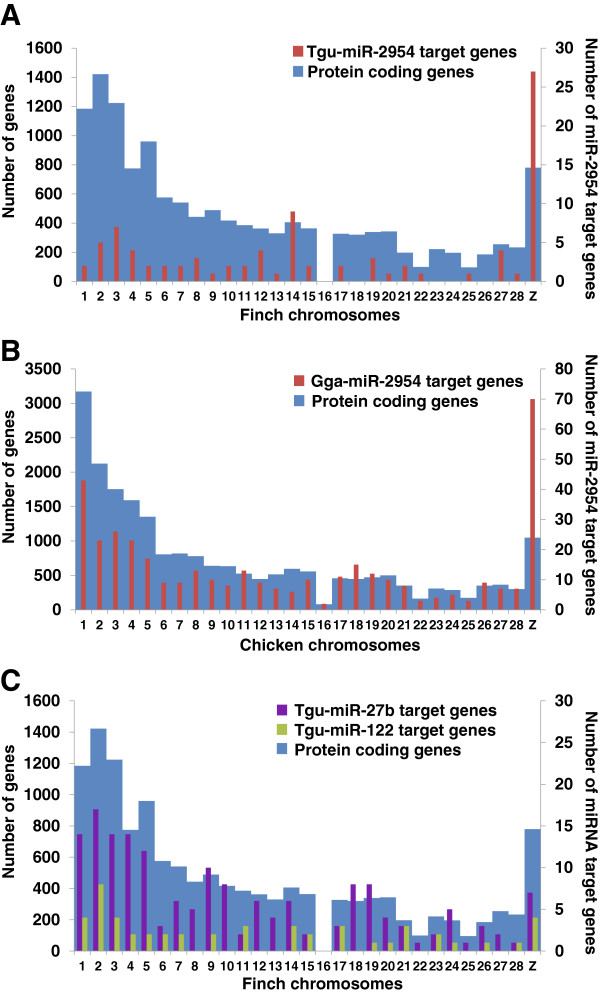
**Z chromosome distribution bias of predicted targets for miR-2954. **(**A**) Distribution of predicted targets for miR-2954 among zebra finch chromosomes. (**B**) Distribution of predicted targets for miR-2954 among chicken chromosomes. Note, in both zebra finches and chickens, miR-2954 targets exhibited a strong bias towards Z chromosome-encoded genes. (**C**) Distribution of predicted targets for miR-27b and miR-122, two other Z chromosome-encoded miRNAs, among zebra finch chromosomes. The distributions of protein coding genes among zebra finch and chicken chromosomes are shown in blue color.

We next performed target prediction analysis using the TargetScan software package [51]. Target analysis predicted approximately 100 putative tgu-miR-2954 target genes, with a strong bias towards Z chromosome-encoded genes (27 of 100, *P < 1e-15*, Fisher′s exact test) (Figure 6A). A similar pattern was also observed for chicken miR-2954, for which 70 of the 403 putative targets were Z chromosome encoded (17.4%, *P < 2.2e-16*, Fisher′s exact test) (Figure 6B), suggesting that the functional preference of miR-2954 for Z-linked genes is conserved among avians. A significantly larger number of miR-2954 target genes were predicted in chickens (WASHUC2) than in zebra finches, probably reflecting the incomplete annotation of the 3'-UTR sequences in the current zebra finch genome assembly (taeGut3.2.4). In contrast, no enrichment of Z chromosome-encoded target genes was found for other zebra finch Z chromosome-encoded miRNAs such as miR-27b and miR-122 (Figure 6C). Among the putative tgu-miR-2954 targets were several Z-linked genes encoding proteins with particular functions in the nervous system, including Ca^2+^/calmodulin-dependent protein kinase IV (CaMKIV), SCAMP1, and SMARCA2. Genes encoding proteins in the guanine nucleotide exchange signaling pathways (e.g., RICTOR, DelGEF-interacting protein 1, Brefeldin A-inhibited guanine nucleotide-exchange protein 3, RAB40C, developmentally-regulated GTP-binding protein 2, and MCF2) were prominently represented as well. In addition, many putative miR-2954 targets were key regulators of transcription, including TLE4, GPBP1, PSIP1, SMARCA2, HMGB1, TNIP3, ZCCHC4, EAPP, RQCD1, MLF1, STAT3, ATF6, EZH1, and DEDD (Additional file 13).

## Discussion

Here we report a comprehensive annotation and analysis of 193 zebra finch miRNAs expressed in four different tissues of both sexes. While the majority of the 193 identified miRNAs are evolutionarily conserved, relatively large fractions of them are zebra finch-specific (10%) or avian-specific (15%). The list of zebra finch specific miRNAs may be even longer, as approximately 40% of the total sequence reads obtained from our small RNA libraries failed to map to the genome, probably due to gaps in the current genome assembly. Conversely, genomes of other species that we searched may have gaps as well, thus we cannot rule out the possibility that some avian and/or zebra finch specific miRNAs described here may have undiscovered homologs in other species. Several zebra finch specific miRNAs showed tissue specific expression. For example, miR-2963 was detected in the brain, miR-2997 in liver, and the novel miRNA 3 in heart (Additional file 14). However, most of the zebra finch specific miRNAs were expressed at low levels, indicating that their expression is restricted to specific cell types or that they have not yet been widely incorporated into gene regulation networks. Further investigation will be needed to determine if these avian or zebra finch specific miRNAs play roles in zebra finch specific features.

Recently, Gunaratne et al. reported the annotation of 155 miRNAs expressed in the auditory forebrain region of zebra finches [35]. Among these miRNAs, 140 are present in our set of miRNAs. Our dataset contains an additional 53 miRNAs, many of which show tissue specific expression in heart, liver, and muscle (Additional files 2 and 15). This is not unexpected as we sequenced four tissues, whereas only auditory forebrain was sequenced by Gunaratne et al. Although the source materials used in the two studies were different and the criteria for miRNA annotation were slightly different, the large overlap between the two datasets provides additional confidence with respect to the identification of these miRNAs. Combining the two sets of data brings the total number of zebra finch miRNAs to 208. Many of these miRNAs show enriched expression in the brain, including several (miR-25, miR-192, miR-124, miR-129, and miR-92) that are regulated in the auditory forebrain by song exposure [35], indicating that miRNAs may play important roles in song behavior and neural plasticity.

### miRNA sequence heterogeneity

We did not observe significant A-to-I editing in mature zebra finch miRNAs except for miR-24. Notably, several mammalian miRNAs with well-characterized A-to-I editing sites (e.g., miR-376a, miR-376c, miR-379, miR-381, miR-411, miR-421, and miR-589, [12,48,49]) do not appear to have homologs in zebra finches, hinting that A-to-I editing might be more restricted to mammals. Instead, we observed internal nucleotide changes at the uridine of a GGU motif occurring at a relative high frequency. Our average base call sequencing error rate was <0.5% (calculated based on spiked-in synthetic internal controls), comparable to sequencing error rates observed by others [49]. The rate of observed nucleotide changes at the GGU motif was as high as 14% (in miR-22). Thus it is unlikely that these changes were due to sequencing errors. We also examined each genomic locus for miRNAs known to have multiple genomic loci, and ruled out the possibility that they were due to genetic variations. We cannot rule out the possibility that these changes were due to single nucleotide polymorphisms (SNPs), as we currently know very little about SNPs in zebra finches. However, we would expect that the rate of nucleotide changes generated by SNPs would be much higher than we observed. To our knowledge, internal nucleotide changes at the uridine of a GGU motif has not been reported previously. Some of the GGU motifs are within seed sequences of mature miRNAs (e.g., members of the let-7 family). Nucleotide changes at these sites can change miRNA binding specificity, and subsequently, impact gene expression and related cellular processes. Further analysis will bring better understandings of biosynthesis mechanisms and potential biological functions of this type of internal nucleotide change.

Other types of sequence heterogeneity including untemplated 3' terminal extensions and length variations are far more prevalent than internal nucleotide changes. miRNA isoforms with 3' untemplated extensions account for ~15% of all miRNA reads. miRNA 3' untemplated A or U extensions have been widely reported by others in multiple animal species [12,13,52-56]. These events seem to be miRNA specific, as some miRNAs are more frequently extended than others. Interestingly, many of the zebra finch miRNAs showing high frequencies of 3' untemplated extensions are also frequently extended in other species (Additional file 10, [11,57,58]), hinting that mechanisms governing the extension events and their potential functions are likely to be conserved. Although investigation of 3' untemplated extension is still at an early stage, 3' extension appears to be biologically regulated [11] and to affect biological function. Emerging evidence suggests that 3' untemplated extensions provide a post-transcriptional mechanism to regulate miRNA stability and efficiency of target repression [57-59].

### miR-2954 as a sex specific gene regulator in avian species

Despite extensive searching, we did not find sequence homologs of miR-2954 outside of the avian taxon. An extensive search among transcripts of crocodile and 11 bird species conducted by Gunaratne et al. found miR-2954 in several bird species but not in crocodile [35]. Given that the genomes and transcriptomes searched in these studies are not complete, we cannot rule out the possibility that miR-2954 has as yet unidentified homologs in other species. However, its expression in chickens and zebra finches is now supported by three independent studies including ours [32,35,50]. The male-biased expression of miR-2954 in chicken embryos [50] and in various zebra finch tissues suggests that it plays a role in sexually dimorphic animal development and function in avian species.

Animals adapt different dosage compensation mechanisms to balance the expression of sex chromosome genes between the two sexes and to balance the expression between sex chromosomal genes and autosomal genes [26,60]. In avian species, females are heterogametic with one Z and one W chromosome, and males are homogametic with two Z chromosomes. Both the chicken and zebra finch lack a chromosome-wide dosage compensation mechanism, and many Z chromosome genes exhibit higher expression in males than in females [61-67]. While the male to female (M:F) expression ratios of most Z-linked miRNAs are close to 2, reflecting the copy number relationship, miR-2954 exhibits M:F expression ratios ranging from 6 to 20 (based on read counts) or 6 to 40 (based on qRT-PCR) in various tissues, which cannot be explained by the 2:1 difference in gene copy numbers. Clearly, additional male-biased factors contribute to the regulation of miR-2954 expression in various tissues.

In both chickens and zebra finches, dosage compensation can occur locally in a gene-specific manner [65-67]. In chickens, this is exemplified by dosage compensation mediated by the *MHM* (male hypermethylated) locus on nearby genes on the Z chromosome [26,68,69]. However, the zebra finch genome does not appear to contain the *MHM* locus [66], suggesting the existence of other sex specific dosage compensation mechanisms. The male-biased expression of miR-2954 and its preferential targeting of Z-chromosome genes may provide a novel dosage compensation mechanism at the post-transcriptional level. A miRNA-mediated gene-specific mechanism for sex chromosome gene regulation would offer flexibility in response to specific developmental and functional needs.

Both song behavior and the underlying neural circuit are highly sexually dimorphic in zebra finches. The song system nuclei HVC [70] and the robust nucleus of the arcopallium (RA), which control motor patterns of song, are considerably larger in males than in females, and Area-X, a forebrain nucleus required for song learning, is a large nucleus in adult males but is almost invisible in adult females [27,71]. The gene regulation network underlying this structural and functional sexual dimorphism is not clear. Among the putative targets of miR-2954, *SCAMP1*, a Z chromosome encoded synaptic vesicle associated protein gene, is known to be expressed at higher levels in HVC and RA in male zebra finches than in females [72]. Recently, Gunaratne et al. reported that miR-2954 is regulated by hearing songs in the auditory forebrain of zebra finches [35], suggesting that in the context of song behavior this miRNA plays important roles in relaying physiological changes to changes in gene expression. Further analysis of miR-2954 expression in the song control circuits, and validation of its target genes will provide a better understanding of its roles in the sexually dimorphic structural and functional development of the zebra finch brain.

## Conclusions

Our results provide a comprehensive miRNA expression atlas of brain, heart, liver, and muscle tissues of both male and female zebra finches. These data significantly enlarge the zebra finch miRNA repertoire, and will serve as a valuable resource for comparative and functional studies for the scientific community. In addition, we report a GGU motif as a potential site for miRNA internal substitution. We also describe male-biased expression of tgu-miR-2954, as well as its Z chromosome biased target relationship, which may point to a novel avian specific dosage compensation mechanism.

## Methods

### Library preparation and sequencing

Four tissues (heart, liver, muscle, and whole brain) from adult male and female zebra finches were collected, and total RNA was isolated using the Trizol method. The RNAs were used for library construction. Briefly, in a total reaction volume of 20 μl, 2 μg total RNA was ligated to 100 pmol adenylated 3′ adapter containing a unique pentamer barcode (App-(Barcode)TCGTATGCCGTCTTCTGCTTGT), 1 μg Rnl2(1–249)K227Q (plasmid for expression of recombinant ligase is available at http://www.addgene.org) in 50 mM Tris–HCl, pH 7.6; 10 mM MgCl_2_; 10 mM 2-mercaptoethanol; 0.1 mg/ml acetylated BSA (Sigma, St. Louis, MO), and 15% DMSO for 16 hours on ice. Following 3′ adapter ligation, 20 bar-coded samples were pooled and products were purified on a 15% denaturing polyacrylamide gel. Small RNAs, measuring 45–50 nt in length, were excised from the gel, eluted, and ligated to 100 pmol 5′ oligoribonucleotide adapter (GUUCAGAGUUCUACAGUCCGACGAUC) in a 20 μl reaction volume using 1 μg Rnl1 RNA Ligase in 50 mM Tris–HCl, pH 7.6; 10 mM MgCl_2_; 10 mM 2-mercaptoethanol; 0.2 mg/ml acetylated BSA; 0.2 mM ATP, and 15% DMSO for 1 h at 37°C. Ligated small RNAs were purified on a 12% polyacrylamide gel, reverse transcribed using SuperScript III Reverse Transcriptase (Invitrogen, Carlsbad, CA), and amplified by PCR using appropriate primers (forward primer: AATGATACGGCGACCACCGACAGGTTCAGAGTTCTACAGTCCGA; RT and reverse primer: CAAGCAGAAGACGGCATACGA). A total of eight libraries were barcoded and sequenced in one lane using Illumina GAII.

### Sequence analysis and miRNA identification

After adapter trimming and removal of orphan reads, reads of 18–32 nt in length were kept for further analysis. For miRNA annotations, we compared sequences to known miRNAs (miRBase 17.0, 04/2011), and identical sequences were identified as homology miRNAs. Sequences were mapped to the zebra finch genome (Ensembl, taeGut3.2.4) permitting no mismatches. Sequences with more than 100 genomic loci were excluded from further analysis. Sequences homologous (with at most one mismatch) to known tRNA/rRNA/ncRNA sequences collected from the NCBI GenBank database were classified as tRNAs/rRNAs/ncRNAs. Small RNAs derived from repeat region/transposable elements were identified by screening the zebra finch genome using the RepeatMasker software. The remaining sequences were used for new miRNA candidate prediction. Flanking genomic sequences of various lengths (60, 80, 100, and 120 nt) of each small RNA mapping locus were extracted and subjected to analysis by the mFold program (http://mfold.rna.albany.edu/) [36] to predict secondary structures. miRNA candidates were identified using the following criteria: (1) presence of hairpin-shaped precursor structures, (2) presence of >10 sequence reads, (3) presence of star sequences originated from the opposite stem of the hairpin structure, and (4) precise 5’ ends among all sequence variants. In addition, sequences homologous to known miRNAs in miRBase, without the star sequence were also accepted. Because the incompleteness of the current genome precluded unambiguous analysis, 16 miRNAs which did not meet criterion 1 but were homologous to known miRNAs were also accepted.

### Sequence conservation analysis

We searched miRBase for known miRNA homologs in 9 species: chicken (galGal3), human (hg18), mouse (mm9), platypus (ornAna1), lizard (anoCar2), X. tropicalis (xenTro3), zebrafish (danRer7), drosophila (dm3), and *C. elegans* (ce10). We also compared zebra finch miRNA sequences to the genomes and ESTs (downloaded from the UCSC genome resources) of the same 9 species to search for homologs with at most 1 mismatch to the query sequence and presence of a hairpin shaped precursor structure. Qualified matches were identified as candidate miRNA homologs in the tested species. According to results based on these criteria, zebra finch miRNAs were classified into groups of zebra finch specific, avian specific, conserved in avian, human, and mouse, conserved in vertebrates, and conserved in all tested species.

### Tissue specificity

We normalized the read counts of individual miRNA species to the total miRNA read number in each tissue to obtain an RPM measurement, ″Reads Per Million reads″. RPM values for each miRNA in all tested tissues were compared. We defined a miRNA as ″highly enriched in one tissue″ if it had a minimum of 100 reads in all tissues, and its expression in one tissue accounted for ≥ 90% of all reads in all tissues combined.

### miRNA sequence variant identification

All sequences passing the qualification filter were used for miRNA variant analysis. Length variants were selected among sequences matched perfectly to the zebra finch genome by comparing their lengths with the canonical miRNA sequences. For variants containing terminal modifications and internal substitutions, the NCBI Blastn program was used to compare all qualified sequence reads with identified miRNAs. Sequences matching multiple miRNAs were excluded to avoid ambiguity. The criteria used for accepting an internal nucleotide change variant included i) a requirement that the total number of sequence reads of a miRNA including its variants was > 100, ii) the number of reads with internal changes represented more than 5% of total reads, and iii) the mismatch occurred at positions at least 2 nucleotides from the 5’ and 3’ termini. A plot of the motif analysis (Figure 4C) was generated with the WebLogo program (http://weblogo.berkeley.edu/[73]).

### Target gene prediction

For target gene prediction, the 3’ UTR sequences of all zebra finch and chicken genes were extracted from the Ensemble database (taeGut3.2.4 and WASHUC2) using the BioMart tool (http://www.ensembl.org/biomart/martview). Genes with 3’ UTR of less than 10 nt or without 3’ UTR were excluded. Since TargetScan (version 6.0, [51]) does not support zebra finch sequences, we made minor modifications to adapt it for target analysis in the zebra finch. Genes containing target sequences within the 3’ UTRs that match perfectly to the 2–8 nt of a miRNA sequence were accepted as putative targets. Enrichment of miRNA targets on the Z chromosome was evaluated by Fisher′s exact test.

### Northern blot and qRT-PCR

Total RNAs were extracted from relevant tissues of adult male and female zebra finches with Trizol reagent (Invitrogen). Total RNAs of chicken, human, and mouse were purchased from Ambion. For Northern blot analysis, 20 μg RNA from each tissue was separated on 20% denaturing gel, transferred to a nitrocellulose membrane, and hybridized to relevant miRNA probes labeled with ^32^P-dCTP at 65°C overnight. Hybridization signals were detected using a phosphorimager. For qRT-PCR, reverse transcription was performed using a custom-designed miR-2954 specific primer and the TaqMan microRNA reverse transcription kit (Applied Biosystems). qRT-PCR was performed in a 20 μL reaction volume containing 1.33 μL cDNA, 1 μL 20× custom designed miR-2954 Taqman probe, and the Taqman universal PCR master mix (Applied Biosystems) using conditions of 10 min at 95°C, followed by 40 cycles of 15 sec at 90°C, 1 min at 60°C. Relative miR-2954 expression was calculated by the ∆ ∆ Ct method as described [74]. GAPDH was used as an internal control for normalizing miR-2954 expression, as no sex-based differential expression of GAPDH has been detected in zebra finches [65]. All qRT-PCR reactions were run in triplicate.

## Additional information

The three novel zebra finch miRNAs have been assigned miRBase IDs as tgu-miR-7643 (novel 1), tgu-miR-7644 (novel 2), and tgu-miR-7645 (novel 3).

## Competing interests

All authors declare that they have no competing interests.

## Authors' contributions

GZL carried out the bioinformatics analysis and participated in writing the paper. MH performed small RNA library construction and sequencing work and participated in writing the paper. MB and GHF helped analyze the data. ZS performed RNA isolation, qRT-PCR, and Northern blot assays. TT participated in designing the work. XCL and XJW designed the study, supervised the experiments and data analysis, and wrote the paper. All authors read and approved the final manuscript.

## Supplementary Material

Additional file 1 A summary of small RNA sequences in 8 libraries. Click here for file

Additional file 2 **Sequences, genomic loci, and expression information of all identified miRNAs.** miRNAs without genomic loci were indicated separately. Click here for file

Additional file 3 Conservation status of all identified miRNAs and their expression reads. Click here for file

Additional file 4 Genomic clusters of zebra finch miRNAs and their conservation status in other species. Click here for file

Additional file 5 miRNA* sequences and their expression information. Click here for file

Additional file 6 Sequences and expression of 3 miR-7*s. Mature miRNAs and corresponding miRNA*s are highlighted by red and blue, respectively. Click here for file

Additional file 7 miRNA precursors with both 5p and 3p arms producing high abundant mature miRNAs. Click here for file

Additional file 8 Sequence reads of tgu-miR-451 and precursor structures of the atypically generated miR-451 in zebra finch, mouse, and human. Click here for file

Additional file 9 **Expression patterns of miRNA clusters in the four tissues.** (A) A heatmap was plotted according to the log2 transformed normalized reads in each tissue. (B) The relative expression of three miRNA clusters in 4 tissues. Click here for file

Additional file 10 miRNA variants with untemplated 3′ terminal modifications. Click here for file

Additional file 11 **Summary of internal nucleotide changes observed among miRNA variants.** (A) The total sequence reads of each nucleotide change type detected in all tissues. (B) The relative ratio of each nucleotide change type in each tissue sample. Click here for file

Additional file 12 Expression information of Z chromosome-encoded miRNAs. Click here for file

Additional file 13 Predicted target genes of tgu-miR-2954. Click here for file

Additional file 14 Expression information of zebra finch specific miRNAs. Click here for file

Additional file 15 **Comparison with the miRNA set from the Gunaratne et al. study.** Fifty-three miRNAs unique in this study are listed in the box. Click here for file
