# Supplementary material for: Genome-wide annotation and analysis of zebra finch microRNA repertoire reveal sex-biased expression
Source: BMC Genomics. 2012 Dec 26;13:727. doi: 10.1186/1471-2164-13-727 (PMC3585881; doi:10.1186/1471-2164-13-727)
Supplement: Additional file 1 — A summary of small RNA sequences in 8 libraries. [file 1471-2164-13-727-S1.pdf]

Additional file 1. Summary of small RNA sequences in 8 samples

Classification	Total	Female				Male			
		Brain	Heart	Liver	Muscle	Brain	Heart	Liver	Muscle
**Raw Illumina GA reads**	23366676	2688206	5596759	969577	3096459	1623849	4606782	516456	4268588
**High quality reads after filtering single read sequences**	19424182	2299762	4752051	783321	2437464	1460197	3819237	450480	3421670
**Non-redundant sequences after filtering single read sequences**	402321	141184	228117	84301	212667	78611	212343	46967	181443
**Reads with perfect match to genome (%)**	11708847 (60%)	1467218 (64%)	2861627 (60%)	439337 (56%)	2019463 (59%)	967322 (66%)	2291015 (60%)	252526 (56%)	1410339 (58%)
**Non-redundant sequences with perfect match to genome (%)**	66541 (17%)	29916 (21%)	44722 (20%)	23695 (28%)	49572 (23%)	13344 (17%)	43545 (21%)	13274 (28%)	45815 (25%)
**Reads homology to miRBase miRNA (%)**	12470982 (64%)	1619412 (70%)	3284065 (69%)	441943 (56%)	1168793 (48%)	1265741 (87%)	2361307 (62%)	329407 (73%)	2000314 (58%)
**Non-redundant sequences homology to miRBase miRNA**	78922	36880	47515	17609	30156	30511	42957	14049	38641
**Reads originated from rRNA/tRNA/other ncRNAs (%)**	4754931 (24%)	427621 (19%)	1028406 (22%)	211652 (27%)	939435 (39%)	35455 (2%)	1035338 (27%)	39073 (9%)	1037951 (30%)
**Reads originated from mRNA (%)**	37401 (0.2%)	2763 (0.1%)	5671 (0.1%)	2478 (0.3%)	6903 (0.3%)	989 (0.1%)	5567 (0.1%)	831 (0.2%)	12199 (0.4%)
**Reads originated from repeats (%)**	85655 (0.4%)	7492 (0.3%)	16568 (0.3%)	4109 (0.5%)	18463 (0.8%)	1018 (0.1%)	19471 (0.5%)	1154 (0.3%)	17380 (0.5%)
**MiRNA species**	193	186	179	164	175	184	180	164	177
