# Supplementary material for: Genome-wide annotation and analysis of zebra finch microRNA repertoire reveal sex-biased expression
Source: BMC Genomics. 2012 Dec 26;13:727. doi: 10.1186/1471-2164-13-727 (PMC3585881; doi:10.1186/1471-2164-13-727)
Supplement: Additional file 6 — Sequences and expression of 3 miR-7*s. Mature miRNAs and corresponding miRNA*s are highlighted by red and blue, respectively. [file 1471-2164-13-727-S6.pdf]

miR-7* on precursor 1 (chrZ:52731917-52731994[+]): 215 reads

```
5'  U        A     A       U      UU  GAU
  GU CUGUGUGG AGACU GUGAUUU GUUGUU  UU   A
  CA GGUAUACC UCUGA CACUAAA CAACAG  AA   A
3'  C        G     -       -      UU  AUC
```

miR-7* on precursor 2 (chr10:13221460-13221527[-]): 11 reads

```
5'   U  A     A       U     U  AU  CU
  CUG GG AGACU GUGAUUU GUUGU GU  GG  C
  GAU CC UCUGA CACUGAA CAACA CA  CC  A
3'   C  G     -       -     C  --  CU
```

miR-7* on precursor 3 (chr28:3838847-3838931[-]): 9 reads

```
5'      CU        A  A   G      U     UG G  U
  GCCUGG  CUGUGUGG AG CUA UGAUUU GUUGU  U UU G
  CGGACC  GACAUACC UC GAU ACUAAA CAACA  G AA U
3'      C-        G  C   -      -     GU G  A
```
