# Supplementary material for: Genome-wide annotation and analysis of zebra finch microRNA repertoire reveal sex-biased expression
Source: BMC Genomics. 2012 Dec 26;13:727. doi: 10.1186/1471-2164-13-727 (PMC3585881; doi:10.1186/1471-2164-13-727)
Supplement: Additional file 8 — Sequence reads of tgu-miR-451 and precursor structures of the atypically generated miR-451 in zebra finch, mouse, and human. [file 1471-2164-13-727-S8.pdf]

A

```
------------stem------------loop-----------------stem--------------------
GCCGGCGGGGAAACCGUUACCAUUACUGAGUUUAGUAAUGGUAACGGUUCUGCCGACGGCCGGCAAGGCGGCA   tgu-miR-451 precursor
||||||||||AAACCGUUACCAUUACUGAGUUUAGU|||||||||||||||||||||||||||||||||||||   1875
||||||||||AAACCGUUACCAUUACUGAGUUUAG||||||||||||||||||||||||||||||||||||||   292
||||||||||AAACCGUUACCAUUACUGAGUUUA|||||||||||||||||||||||||||||||||||||||   454
||||||||||AAACCGUUACCAUUACUGAGUUU||||||||||||||||||||||||||||||||||||||||   73784
||||||||||AAACCGUUACCAUUACUGAGUU|||||||||||||||||||||||||||||||||||||||||   41153
||||||||||AAACCGUUACCAUUACUGAGU||||||||||||||||||||||||||||||||||||||||||   16108
||||||||||AAACCGUUACCAUUACUGAG|||||||||||||||||||||||||||||||||||||||||||   327
||||||||||AAACCGUUACCAUUACUGA||||||||||||||||||||||||||||||||||||||||||||   128
||||||||||AAACCGUUACCAUUACUG|||||||||||||||||||||||||||||||||||||||||||||   3
|||||||||||AACCGUUACCAUUACUGAGUUU||||||||||||||||||||||||||||||||||||||||   3026
||||||||||||ACCGUUACCAUUACUGAGUUU||||||||||||||||||||||||||||||||||||||||   33
|||||||||||||CCGUUACCAUUACUGAGUUU||||||||||||||||||||||||||||||||||||||||   4
||||||||||||||CGUUACCAUUACUGAGUUU||||||||||||||||||||||||||||||||||||||||   82
|||||||||||||||GUUACCAUUACUGAGUUU||||||||||||||||||||||||||||||||||||||||   58
```

B

Zebra finch:

```
   A              GA
GGAA CCGUUACCAUUACU  G
UCUU GGCAAUGGUAAUGA  U
    -              UU
```

Mouse:

```
     AU      GA                 A
UGGGA  GGCGAG  AACCGUUACCAUUACUG G
ACCCU  UCGUUC  UUGGCAAUGGUAAUGAU U
     CG      UC                 U
```

Human:

```
     A        GA                 A
UUGGG AUGGCAAG  AACCGUUACCAUUACUG G
GACCC UAUCGUUC  UUGGUAAUGGUAAUGAU U
     A        UC                 U
```
