# Supplementary figures and images for: Genome-wide annotation and analysis of zebra finch microRNA repertoire reveal sex-biased expression
Source: BMC Genomics. 2012 Dec 26;13:727. doi: 10.1186/1471-2164-13-727 (PMC3585881; doi:10.1186/1471-2164-13-727)

A

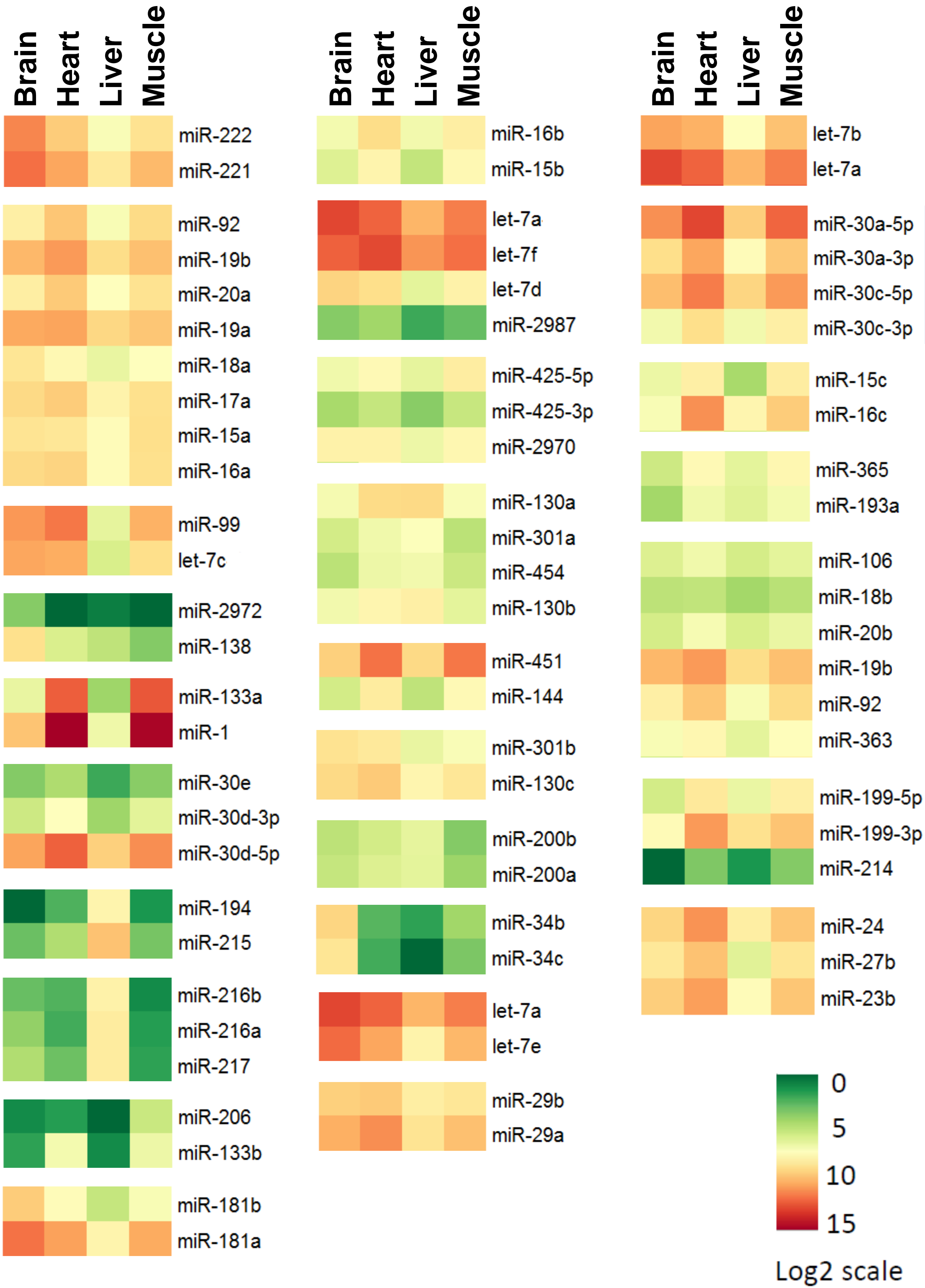

B

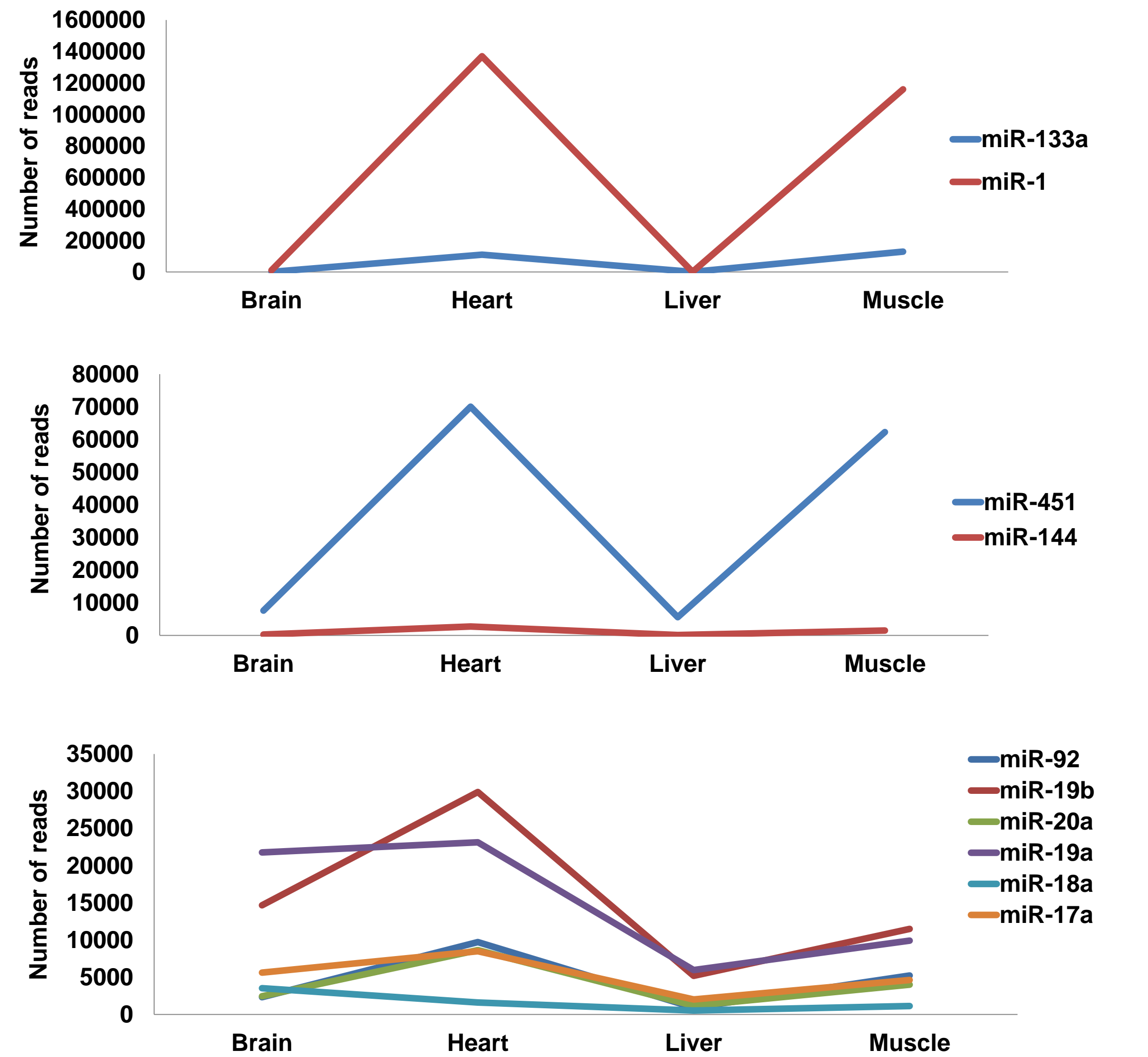

Supplement: Additional file 9 — Expression patterns of miRNA clusters in the four tissues. (A) A heatmap was plotted according to the log2 transformed normalized reads in each tissue. (B) The relative expression of three miRNA clusters in 4 tissues. [file 1471-2164-13-727-S9.pdf]

A

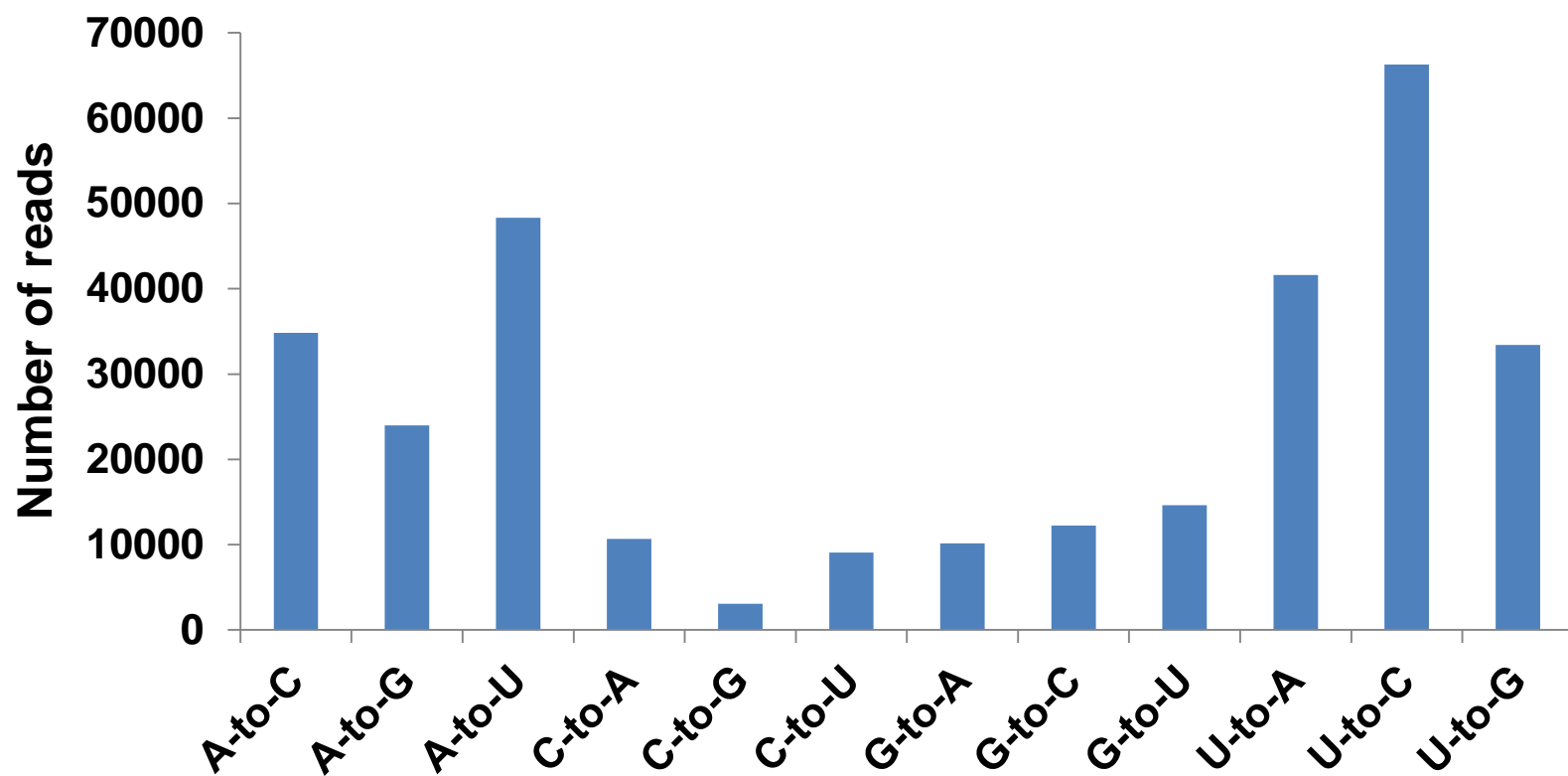

B

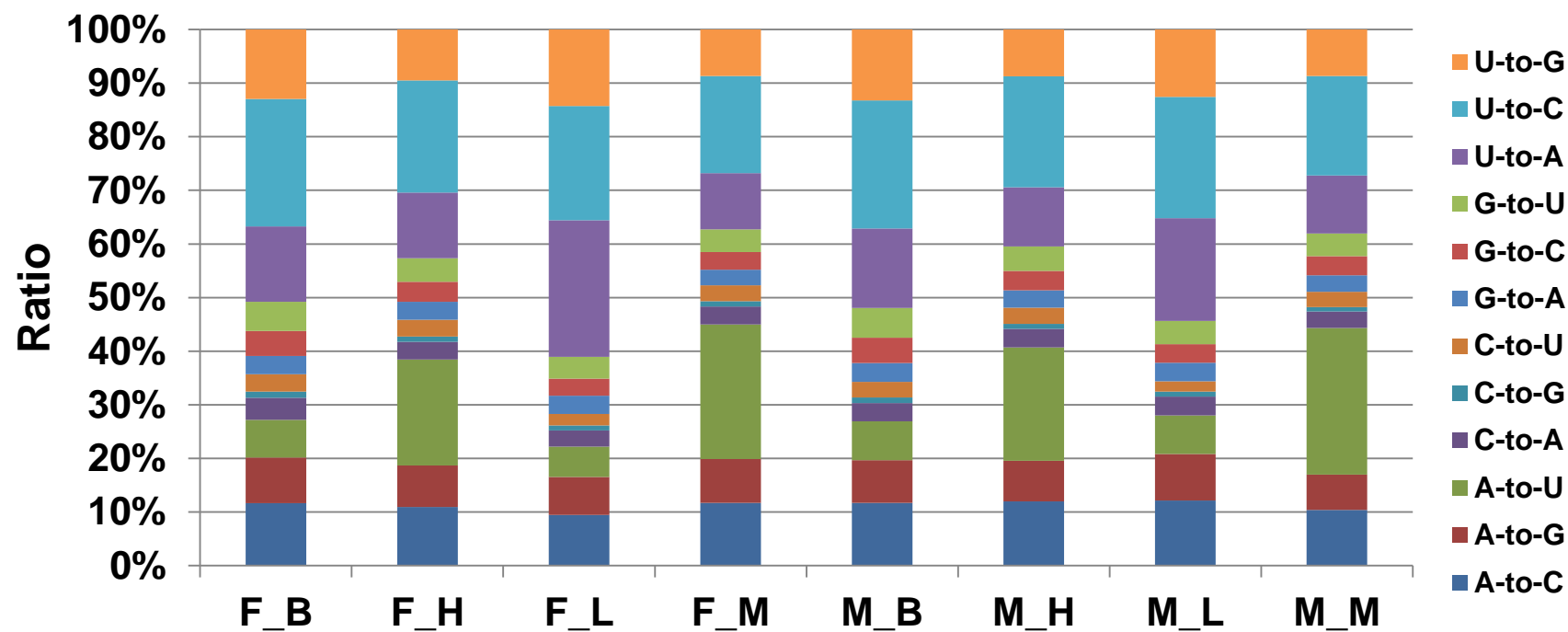

Supplement: Additional file 11 — Summary of internal nucleotide changes observed among miRNA variants. (A) The total sequence reads of each nucleotide change type detected in all tissues. (B) The relative ratio of each nucleotide change type in each tissue sample. [file 1471-2164-13-727-S11.pdf]
