# Supplementary material for: Genome-wide annotation and analysis of zebra finch microRNA repertoire reveal sex-biased expression
Source: BMC Genomics. 2012 Dec 26;13:727. doi: 10.1186/1471-2164-13-727 (PMC3585881; doi:10.1186/1471-2164-13-727)
Supplement: Additional file 15 — Comparison with the miRNA set from the Gunaratne et al. study. Fifty-three miRNAs unique in this study are listed in the box. [file 1471-2164-13-727-S15.pdf]

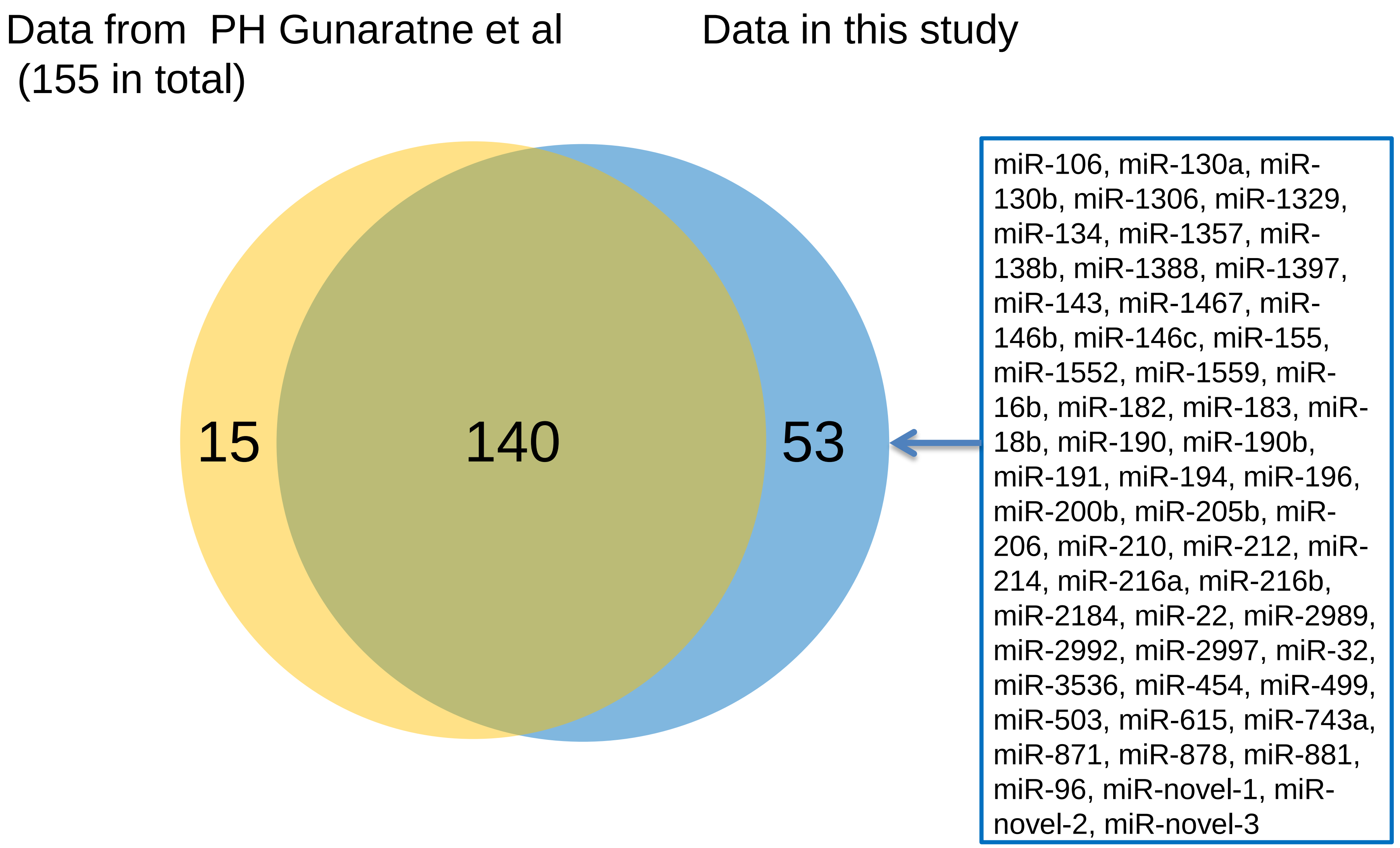
Data from PH Gunaratne et al
(155 in total)
Data in this study
15
140
53
miR-106, miR-130a, miR-130b, miR-1306, miR-1329, miR-134, miR-1357, miR-138b, miR-1388, miR-1397, miR-143, miR-1467, miR-146b, miR-146c, miR-155, miR-1552, miR-1559, miR-16b, miR-182, miR-183, miR-18b, miR-190, miR-190b, miR-191, miR-194, miR-196, miR-200b, miR-205b, miR-206, miR-210, miR-212, miR-214, miR-216a, miR-216b, miR-2184, miR-22, miR-2989, miR-2992, miR-2997, miR-32, miR-3536, miR-454, miR-499, miR-503, miR-615, miR-743a, miR-871, miR-878, miR-881, miR-96, miR-novel-1, miR-novel-2, miR-novel-3
